# Mitophagy-driven mitochondrial rejuvenation regulates stem cell fate

**DOI:** 10.18632/aging.100976

**Published:** 2016-06-13

**Authors:** Alejandro Vazquez-Martin, Chris Van den Haute, Sílvia Cufí, Bruna Corominas-Faja, Elisabet Cuyàs, Eugeni Lopez-Bonet, Esther Rodriguez-Gallego, Salvador Fernández-Arroyo, Jorge Joven, Veerle Baekelandt, Javier A. Menendez

**Affiliations:** ^1^ Cancer Research Group, Latvian Biomedical Research & Study Centre, Riga, Latvia; ^2^ Laboratory for Neurobiology and Gene Therapy, Department of Neurosciences, Katholieke Universiteit Leuven, Leuven, Flanders, Belgium; ^3^ Josep Carreras Leukemia Research Institute, Stem Cell Lab, Barcelona, Spain; ^4^ Molecular Oncology Group, Girona Biomedical Research Institute (IDIBGI), Girona, Spain; ^5^ Department of Anatomical Pathology, Dr. Josep Trueta Hospital of Girona, Girona, Catalonia, Spain; ^6^ Unitat de Recerca Biomèdica, Hospital Universitari Sant Joan, Institut d'Investigació Sanitaria Pere Virgili (IISPV), Universitat Rovira i Virgili, Reus, Spain; ^7^ ProCURE (Program Against Cancer Therapeutic Resistance), Metabolism and Cancer Group, Catalan Institute of Oncology, Girona, Spain

**Keywords:** mitophagy, mitochondria, stem cells, pluripotency, cancer, epigenetics

## Abstract

Our understanding on how selective mitochondrial autophagy, or mitophagy, can sustain the archetypal properties of stem cells is incomplete. PTEN-induced putative kinase 1 (PINK1) plays a key role in the maintenance of mitochondrial morphology and function and in the selective degradation of damaged mitochondria by mitophagy. Here, using embryonic fibroblasts from *PINK1* gene-knockout (KO) mice, we evaluated whether mitophagy is a causal mechanism for the control of cell-fate plasticity and maintenance of pluripotency. Loss of *PINK1*-dependent mitophagy was sufficient to dramatically decrease the speed and efficiency of induced pluripotent stem cell (iPSC) reprogramming. Mitophagy-deficient iPSC colonies, which were characterized by a mixture of mature and immature mitochondria, seemed unstable, with a strong tendency to spontaneously differentiate and form heterogeneous populations of cells. Although mitophagy-deficient iPSC colonies normally expressed pluripotent markers, functional monitoring of cellular bioenergetics revealed an attenuated glycolysis in mitophagy-deficient iPSC cells. Targeted metabolomics showed a notable alteration in numerous glycolysis- and TCA-related metabolites in mitophagy-deficient iPSC cells, including a significant decrease in the intracellular levels of α-ketoglutarate -a key suppressor of the differentiation path in stem cells. Mitophagy-deficient iPSC colonies exhibited a notably reduced teratoma-initiating capacity, but fully retained their pluripotency and multi-germ layer differentiation capacity *in vivo*. *PINK1*-dependent mitophagy pathway is an important mitochondrial switch that determines the efficiency and quality of somatic reprogramming. Mitophagy-driven mitochondrial rejuvenation might contribute to the ability of iPSCs to suppress differentiation by directing bioenergetic transition and metabolome remodeling traits. These findings provide new insights into how mitophagy might influence the stem cell decisions to retain pluripotency or differentiate in tissue regeneration and aging, tumor growth, and regenerative medicine.

## INTRODUCTION

Mitochondrial autophagy, or mitophagy, is a key cellular pathway for mitochondrial quality control that functions to clear mitochondria [1-4]. Because the selective autophagosome-based mitochondrial degradation process eliminates unwanted or dysfunctional mitochondria after cell stress [5-9], abnormal mitophagy has a deleterious impact on cell homeostasis and may lead to cell death, which causally contributes to the pathogenesis of degenerative disorders [10-16]. Although our knowledge of mitophagy in somatic cell physiology is extensive, the role of mitophagy in the physiology of stem cells, which have the unique ability to self-renew and differentiate into various cell types, is less understood. Thus, while mitophagy is believed to play a pivotal role in stem cell functions during aging, tissue regeneration, and cancer [17-24], our current understanding on how mitophagy can sustain the archetypal properties of stem cells is rudimentary.

Mitochondria appear to play crucial roles during stemness factor-mediated nuclear reprogramming of somatic cells into induced pluripotent stem cells (iPSCs), a convenient “in a dish” model that allows a comprehensive understanding of stem cell biology. Functional metamorphosis of somatic oxidative phosphorylation into glycolytic metabolism plays a causal role in enabling the reprogramming process of acquisition and maintenance of stemness to occur [25-35]. It is also apparent that the intrinsic metabolic demands that drive reprogramming to stemness involve substantial structural mitochondrial reorganization, transforming mitochondria into a cristae-poor, immature phenotype [36-45]. Paradoxically, the establishment of induced pluripotency requires a transient and early energy-demanding metabolic state characterized by increased mitochondrial oxidative phosphorylation and hyperactive mitophagy [46, 47]. Because the unique metabolic state required to achieve cell plasticity is accompanied by significant temporal changes in mitochondrial function, composition, structure, and maturation, it might appear elementary to suggest that mitophagy is a prerequisite of induced pluripotency. Nonetheless, recent studies have shed light on how interlinked processes critical for mitochondrial health, including mitochondrial fragmentation and mito-chondrial fission/fusion, significantly alter the efficiency and speed of induced pluripotency [48-51], but little information is available on the role of mitophagy in the acquisition and maintenance of stemness.

PTEN-induced putative kinase 1 (*PINK1*) encodes a key mitochondrial protein that specifically identifies and commits mitochondria to degradation via selective autophagy [52-63]. Using embryonic fibroblasts from *PINK1* gene-knockout (KO) mice, we here tested the hypothesis that mitophagy is a pivotal mechanism of cell-fate plasticity by converting functionally mature mitochondrial networks into immature states and vice versa during nuclear reprogramming to stemness and commitment to differentiation, respectively. By examining the ability of mitophagy to causally modulate cell fate decisions during the entry to and exit from pluripotency, we have identified a hitherto unrecognized role of the mitophagy pathway as a critical mitochondrial switch that directs bioenergetic transition and metabolome remodeling traits to ultimately determine the efficiency and quality of nuclear reprogramming and stemness transition in somatic cells.

## RESULTS

### PINK1-mediated mitophagy is necessary for efficient nuclear reprogramming of somatic cells into iPSCs

Because the initial stages of reprogramming trigger a stress response involving repression of mitochondrial functions and oxidative stress [36-45, 66], we hypothesized that the critical ability of PINK1 to identify and selectively trim impaired mitochondria from the mitochondrial network might determine the efficiency of reprogramming. To test whether *PINK1*-KO mouse embryonic fibroblasts (MEFs) constitute a useful model to dissect the role of mitophagy in the establishment of induced pluripotency, we first mimicked mitochondrial damage by experimentally depolarizing mitochondria with the uncoupler carbonyl cyanide *m*-chlorophenylhydrazone (CCCP) and then monitoring loss of MitoTracker staining after mitophagy stimulation [62, 67]. Collectively, our findings show that PINK1 is necessary to efficiently drive the mitophagic digestion of damaged mitochondria (Supplemental data; Fig. supplemental 1).

**Figure 1:**
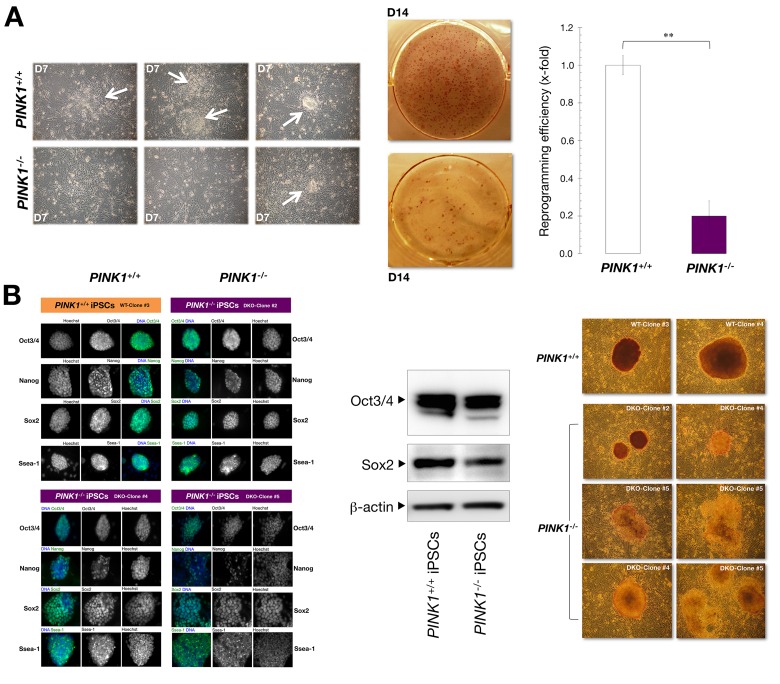
Mitophagy deficiency is a reprogramming barrier (**A**) Early passage *PINK1^+/+^* and *PINK1^−/−^* MEFs were transduced with retroviral vectors encoding for Oct4, Sox2, and Klf4 and cultured in ES medium. Phase-contrast microphotographs of representative *PINK1^+/+^* and *PINK1^−/−^* MEFs at day 7 (D7) after the initial transduction with OSK are shown (white arrows indicate emerging iPSC-like colonies). Representative photographs of colonies of AP-stained OSK-transduced *PINK1^+/+^* and *PINK1^−/−^* MEFs. The number of AP^+^ colonies was counted 14 days after the initial infection and represent reprogramming efficiency relative to *PINK1^+/+^* MEFs (x-fold) (n=6 for each condition). **, P<0.01. (**B**) Individual iPSC-like colonies were randomly selected from each *PINK1* subtype, cultured on 6-well plates coated with MEF feeder layers, and stained either for AP activity (*left*) or with antibodies against Oct3/4, Nanog, Sox2, and Ssea-1 (*right*), as indicated in the “Methods” section. Nuclear staining was performed with Hoechst 33258. Panel depicts representative images of iPSC colonies that were captured using different channels for Oct3/4 (*green*), Nanog (*green*), Sox2 (*green*), Ssea-1 (*green*), or Hoechst 33258 (*blue*), as specified. Representative Western blots for Oct3/4 and Sox2 protein expression in *PINK1^+/+^*- *PINK1^−/−^*-iPSCs are shown (*middle*; n = 2).

We then used *PINK1^−/−^* MEFs to explore whether PINK1-mediated mitophagy might constitute part of the molecular roadmap facilitating reprogramming. To do this, we compared iPSC generation in early-passage *PINK^+/+^* and *PINK^−/−^* MEFs using a three-factor induction protocol (*Oct4*, *Sox2*, and *Klf4*, hereafter referred to as OSK). We transduced MEFs with OSK at a 1:1:1 ratio on day 0 and repeated the transduction up to four times (one infection every 12 h using the same batch of all three retroviruses), after which the regular media was replaced with standard mESC media supplemented with the knockout serum replacement (KSR). As early as 7 days after transduction, clearly recognizable flat, packed, tight colonies characteristic of ES-like cells appeared in OSK-transduced *PINK1^+/+^* MEF cultures (Fig. 1A). Conversely, OSK-transduced *PINK1^−/−^* MEFs mostly failed to display the typical compact ES cell colony morphology (Fig. 1A). Indeed, using a parallel live cell-imaging 96-well-plate-based screening assay to rapidly assess the expression of the pluripotency-associated surface marker Ssea-1 during reprogramming, we found that the appearance of Ssea-1^+^ clusters was delayed by 3-4 days in *PINK1^−/−^* MEFs compared with *PINK1^+/+^* isogenic counterparts (data not shown). We combined the observations of ES cell-like morphological changes (e.g., defined boundaries and high nucleus-to-cytoplasm ratio within individual cells) with alkaline phosphatase (AP) activity, a commonly used pluripotency indicator, to quantify *bona fide* iPSC colonies. From 50,000 *PINK1^+/+^* MEFs transduced, 150±10 colonies were AP-positive at day 14 after transduction, resulting in an iPSC generation efficiency of 0.3% (Fig. 1A). In contrast, only 30±4 colonies were generated from an equivalent number of *PINK1^−/−^* MEFs, equivalent to an iPSC generation efficiency of 0.06% (Fig. 1A). Concerning the transduction efficiency, we did not observe any significant differences between the two groups (data not shown), thus confirming that the observed decrease in reprogramming efficiency is due to the absence of *PINK1*. These findings show that loss of PINK1-dependent mitophagy is sufficient to dramatically decrease the speed and efficiency of nuclear re-programming.

### Loss of PINK1-mediated mitophagy destabilizes the undifferentiated state in iPSCs

To verify that loss of PINK1-driven mitophagy dampens the reprogramming process, we picked random colonies from the previous experiment and established *PINK1^+/+^*-iPSC and *PINK1^−/−^*-iPSC clonal cell lines on pre-seeded MEF feeder layers, which are known to encourage induced stem cells to remain in an undifferentiated state. Immunofluorescence staining analysis of early-passage iPSCs failed to reveal major differences in the expression level of Oct3/4, Nanog, Sox2, and Ssea-1 of individual cells within *PINK1^−/−^*- and *PINK1^+/+^*-iPSC colonies (Fig. 1B, right). Indeed, immunoblotting procedures demonstrated a similar expression profile of the pluripotency markers Oct3/4 and Sox2 in early-passage *PINK1^−/−^*- and *PINK1^+/+^*-iPSCs (Fig. 1B, middle). Both early- and late-passage *PINK1^+/+^*-iPSC colonies were large and well-rounded and stained strongly for AP, reflective of a high percentage of non-differentiated cells (Fig. 1B). The majority of relatively late-passage *PINK1^−/−^*-iPSC colonies, however, were slightly smaller upon colony expansion, with very few showing a smooth, circular and distinct edge, and in many cases displaying irregular morphologies and undefined edges. Indeed, late-passage *PINK1^−/−^*-iPSC colonies tended to rapidly lose AP activity and failed to stably retain the compact colony morphology typical of undifferentiated iPSCs [50, 68] (Fig. 1B, right).

Given that the apparent destabilization of the undifferentiated state of *PINK1^−/−^*-iPSCs occurred without drastic changes in the expression of several pluripotency markers, we explored whether loss of PINK1-driven mitophagy significantly altered the well-known ability of nuclear reprogramming to transform the mitochondrial infrastructure and induce iPSC-associated bioenergetic transition and metabolome remodeling traits [30].

### Mitophagy deficient-iPSCs cannot “rejuvenate” the morphological characteristics of the mitochondria network

**Figure 2:**
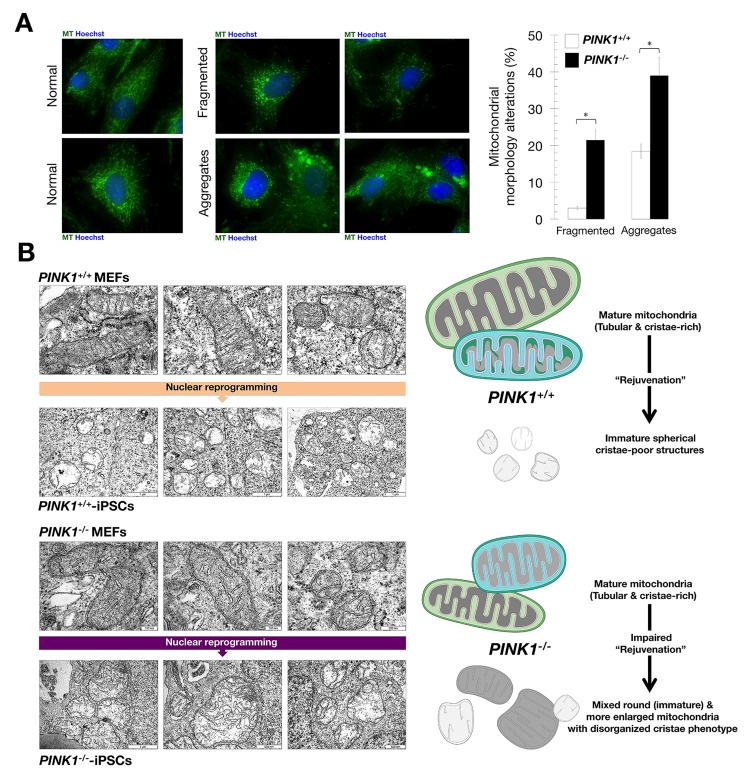
Mitophagy deficiency impedes the rejuvenation of mitochondria networks in iPSCs (**A)** Loss of *PINK1* induces moderate mitochondrial fragmentation and aggregation in MEFs (MT: MitoTracker). Bar chart depicts the average percentages of cells showing fragmentation and/or aggregation (n=3). *, P<0.05. (**B)** Representative TEM images of mitochondria in all cell lines. *PINK1^+/+^* and *PINK1^−/−^* MEFs display a preponderance of tubular and cristae-rich mature mitochondria. *PINK1^+/+^* iPSCs display a preponderance of “rejuvenated” spherical cristae-poor immature mitochondria, whereas *PINK1^−/−^* iPSCs display an impaired “rejuvenation” characterized by an assortment of mitochondrial configurations including round (immature) and more enlarged mitochondria with disorganized cristae

We first confirmed that loss of *PINK1* induces a moderate mitochondrial fragmentation in MEFs [52] as well as a more prominent accumulation of mitochondrial aggregates due to impaired mitophagy (Fig. 2A). Given that cell reprogramming leads to mitochondrial structural and functional alterations described as “rejuvenation” [36-42, 44, 45, 66], we used transmission electron microscopy (TEM) to examine whether the decreased capacity of *PINK1^−/−^*-iPSCs to maintain their undifferentiated state involves alterations in the morphology of the mitochondrial network. Although we could detect a small decrease in the length and area of mitochondria in *PINK1^−/−^* MEFs, together with an increase in the number of mitochondria per cell (Fig. 2B), the majority of mitochondria in both MEF populations had a similar morphology characterized by mature mitochondrial networks with tubular structures and densely-packed cristae (Fig. 2B).

*PINK1^+/+^*-iPSC mitochondria exhibited a dramatically decreased long diameter and increased short diameter relative to *PINK1^−/−^* MEFs (Fig. 2B). Interestingly, though nuclear reprogramming of *PINK1^+/+^*-iPSCs led to the acquisition of an immature mitochondrial phenotype characterized by a rounded morphology with sparse cristae, it was noteworthy that reprogramming failed to fully reset the mitochondrial morphology of *PINK1^−/−^* MEFs to a *bona fide* embryonic-like state. Con-sequently, *PINK1^−/−^* iPSCs accumulated larger, irregular mitochondria containing different inclusions and more cristae (Fig. 2B). These findings, altogether, strongly suggest that mitophagy deficient-iPSCs fail to fully rejuvenate the morphological characteristics of the mitochondrial network.

### Loss of PINK1-driven mitophagy impairs the bioenergetic transition associated with nuclear reprogramming

Mitochondrial rejuvenation is a key mechanism to protect cells from reprogramming factor-induced oxidative stress and reactive oxygen species (ROS) accumulation, a well-known roadblock to re-programming [36-42, 44, 45, 66]. We therefore speculated that blockade of PINK1-driven mitophagy might lead to a detrimental accumulation of ROS during the initial stages of reprogramming (Supplemental data; Fig. supplemental 2). Only when forced expression of c-Myc, which can override the cell cycle checkpoints imposed in response to ROS accumulation [69-71], was combined with the exogenous addition of vitamin C *PINK1^−/−^* MEFs reached reprogramming efficiencies equivalent to those observed in OSK-transduced *PINK1^+/+^* MEFs in the absence of this antioxidant (Supplemental data; Fig. supplemental 2). Because the incapacity of the ROS scavenger and epigenetic regulator vitamin C [72-75] to fully bypass the reprogramming roadblock imposed by the loss of *PINK1* suggested that mitophagy-driven remodeling of the mitochondria network into an immature state might constitute a critical barrier during somatic reprogram-ming, we performed a multimodal metabolic characterization of *PINK1^−/−^* and *PINK1^+/+^* iPSCs at the level of cellular bioenergetics and intracellular metabolome.

**Figure 3:**
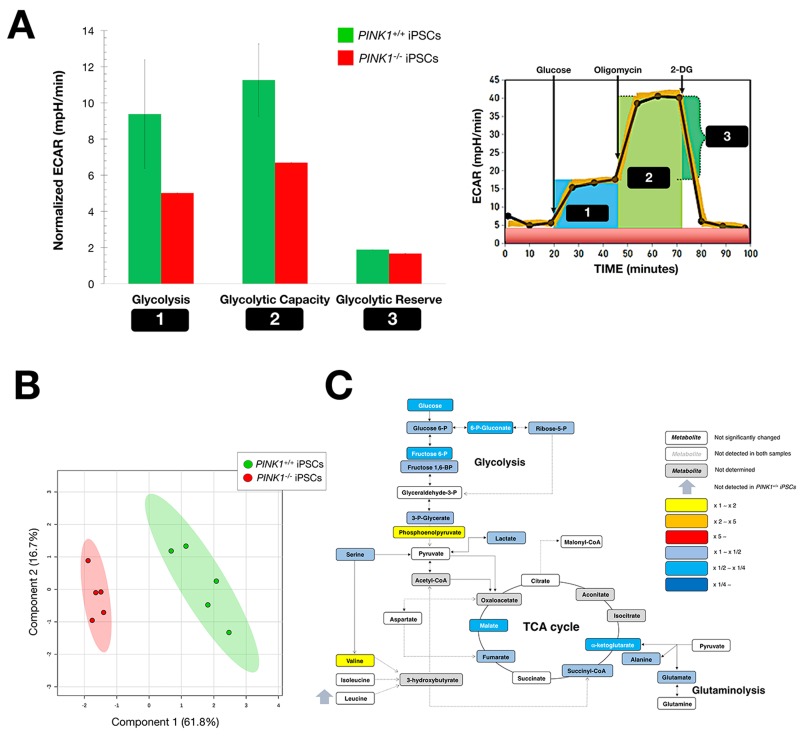
Mitophagy deficiency impairs the bioenergetic transition associated with nuclear reprogramming (**A**) Calculated glycolysis (maximum rate measurement before oligomycin injection – Last rate measurement before glucose injection [1]), glycolytic capacity (maximum rate measurement after oligomycin injection – Last rate measurement before glucose injection [2]), and glycolytic reserve (glycolytic capacity – glycolysis [3]) were calculated with normalized ECAR values in *PINK1^+/+^*- and *PINK1^−/−^*-iPSCs (n = 2). (**B**) Two-dimensional PLS-DA models to view the separation of the two groups (*PINK1^+/+^*- and *PINK1^−/−^*-iPSCs) following GC-EI-QTOF-MS-based metabolomic profiling. (**C**) Metabolites in *PINK1^+/+^*-iPSCs were extracted and quantitatively analyzed by GC-EI-QTOF-MS and compared with metabolites from *PINK^−/−^*-iPSCs (n = 2). Significantly increased and decreased metabolites are shown using yellow-red and light blue-dark blue color scales, respectively (see also Table 1).

First, a well-validated extracellular flux technology was employed to establish functional monitoring of cellular bioenergetics in *PINK1^−/−^* and *PINK1^+/+^* iPSCs. Measurement of the extracellular acidification rate (ECAR) enabled real-time assessment of the glycolytic phenotype associated with iPSCs (Fig. 3A). Sequential supplementation of the glycolytic fuel glucose, the ATP synthase complex V mitochondrial inhibitor oligo-mycin, and the competitive inhibitor of glucose 2-deoxy-glucose (2-DG), dissected key parameters of glycolytic function, including glycolysis (i.e., the ECAR rate reached by iPSCs after the addition of saturating amounts of glucose), glycolytic capacity (i.e., the maximum ECAR rate reached upon blockade of oxidative phosphorylation), and glycolytic reserve (i.e., the capability of iPSCs to respond to an energetic demand). *PINK1^−/−^*-iPSCs were found to be significantly less glycolytic than *PINK1^+/+^*-iPSCs (Fig. 3A), suggesting that the reduced capacity of mitophagy deficient-iPSCs to efficiently drive mito-chondrial metamorphosis translate into a reduced capacity to bioenergetically transitioning from somatic cellular respiration to glycolysis during iPSC derivation [30].

### Loss of PINK1-driven mitophagy impairs the metabolome remodeling associated with nuclear reprogramming

We utilized our recently developed targeted metabolomics platform coupling gas chromatography with quadrupole time-of-flight mass spectrometry and an electron impact source (GC-EI-QTOF-MS), which allows the simultaneous measurement of selected metabolites representative of the catabolic and anabolic status of key metabolic nodes. These metabolites include not only representatives of glycolysis and the mitochondrial tricarboxylic acid (TCA) cycle, but also other biosynthetic routes such as pentose phosphate pathway, amino acid metabolism and *de novo* fatty acid biogenesis [76, 77]. Metabolite-based clustering obtained by partial least squares-discriminant analysis (PLS-DA) model revealed a clear and significant separation between *PINK1^−/−^*-iPSCs and *PINK1^+/+^*-iPSCs in two-dimensional (2D) score plots (Fig. 3B). Profiling of the intracellular metabolome supported a *PINK1^−/−^* iPSCs signature distinct from the *PINK1^+/+^* iPSCs counterpart, which apparently involved a notable decrease in a majority of the measured glycolysis- and TCA-related biochemicals (Fig. 3C).

**Figure 4:**
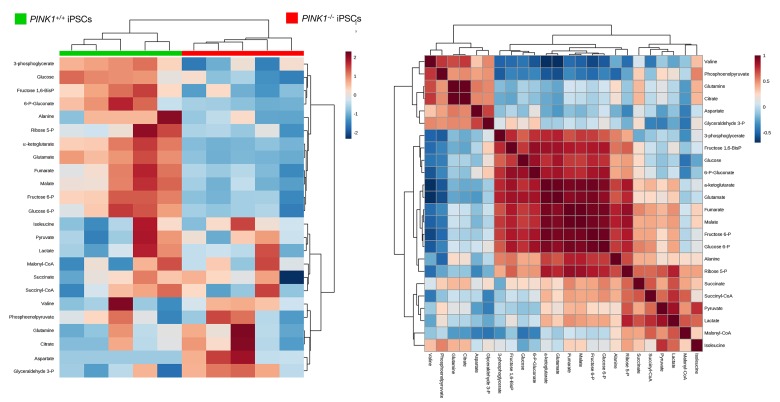
Mitophagy deficiency impairs the metabolome remodeling associated with nuclear reprogramming *Left*. Heatmap to view the agglomerative hierarchical clustering of the *PINK1^+/+^*- and *PINK1^−/−^*-iPSCs groups analyzed with MetaboAnalyst's data annotation tool [103]. Rows: metabolites; columns: samples; color key indicates metabolite expression value (blue: lowest; red: highest). *Right*. Heatmap of correlations between iPSCs metabolites. Each square represents the Spearman's correlation coefficient between the metabolite of the column with that of the row. Metabolite order is determined as in hierarchical clustering using the distance function 1-correlation. Self-self correlations are identified in dark brown.

**Figure 5:**
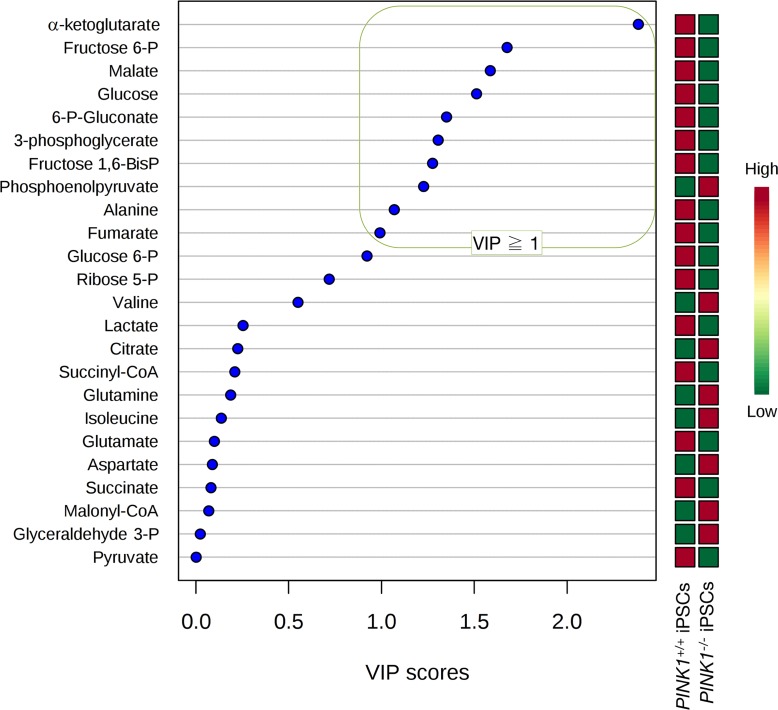
α-ketoglutarate is the most impacted metabolite in mitophagy deficient-iPSCs VIP rank-score of quantified metabolites in the *PINK1^+/+^*- and *PINK1^−/−^*-iPSCs groups. Green box indicates metabolites that achieved VIP scores above 1.0.

Heatmap visualization, commonly used for unsupervised clustering, likewise revealed distinct segregation of metabolites in *PINK1^−/−^*-iPSCs and *PINK1^+/+^*-iPSCs groups, pointing to an altered metabolic signature associated with the loss of *PINK1*-dependent mitophagy in iPSCs (Fig. 4). Unsupervised hierarchical clustering of all pairwise comparisons among individual metabolites revealed several “hot spots” of highly correlated metabolites in a correlation matrix (Fig. 4). When VIP scores ≥ 1 in the PLS-DA model were used to maximize the difference of metabolic profiles between *PINK1^−/−^*- and *PINK1^+/+^*-iPSCs, the TCA metabolite α-ketoglutarate was the metabolite majorly impacted in mitophagy-deficient *PINK1^−/−^*-iPSCs (Fig. 5). Quantitative assessment of metabolite concentrations confirmed that *PINK1^−/−^*-iPSCs significantly accumulated > 3-fold less α-ketoglutarate than *PINK1^+/+^*-iPSCs (Table 1), suggesting that the reduced capacity of mitophagy deficient-iPSCs to efficiently drive mitochondrial metamorphosis during nuclear reprogramming translate into a reduced capacity to achieve the embryonic stem cell-like metabolome that characterizes generated iPSCs.

**Table 1:** Concentration (in μM/mg protein) and fold-change of bioenergetic metabolites in *PINK1^−/−^*- vs *PINK^+/+^*-iPSCs cells

Metabolite	*PINK1^+/+^ iPSCs*	*PINK1^−/−^ iPSCs*	Fold-change
3-phosphoglycerate*	136.079 ± 16.415	77.173 ± 15.581	−1.76
6-P-Gluconate*	11.045 ± 3.159	5.715 ± 0.397	−1.93
α-ketoglutarate*	0.364 ± 0.111	0.116 ± 0.005	−3.14
Alanine	2.435 ± 1.029	1.415 ± 0.335	−1.72
Aspartate	102.883 ± 11.692	106.727 ± 3.873	1.04
Citrate	4.533 ± 0.531	5.104 ± 1.146	1.13
Fructose 1,6-BisP*	144.329 ± 29.863	80.327 ± 20.233	−1.80
Fructose 6-P*	12.079 ± 2.511	5.499 ± 0.759	−2.20
Fumarate*	1.814 ± 0.389	1.130 ± 0.116	−1.61
Glucose*	40.668 ± 7.493	20.546 ± 6.196	−1.98
Glucose 6-P*	9.085 ± 1.600	5.877 ± 0.587	−1.55
Glutamate*	338.181 ± 2.622	322.962 ± 0.710	−1.05
Glutamine	5.122 ± 0.630	5.643 ± 1.159	1.10
Glyceraldehyde 3-P	54.602 ± 5.824	55.356 ± 7.668	1.01
Isoleucine	24.623 ± 7.773	25.745 ± 5.453	1.05
Lactate	212.007 ± 24.712	182.187 ± 18.347	−1.16
Leucine*	ULOQ	4.693 ± 1.384	-
Malate*	10.379 ± 3.172	4.824 ± 0.693	−2.15
Malonyl-CoA	24.615 ± 3.858	25.290 ± 2.881	1.03
Phosphoenolpyruvate*	0.315 ± 0.124	0.541 ± 0.134	1.72
Pyruvate	2.245 ± 0.946	2.136 ± 0.443	−1.05
Ribose 5-P*	6.136 ± 1.768	4.286 ± 0.499	−1.43
Serine*	36.239 ± 1.181	22.870 ± 0.448	−1.59
Succinate	35.714 ± 3.785	34.638 ± 5.498	−1.03
Succinyl-CoA	1.142 ± 0.296	1.044 ± 0.341	−1.09
Valine*	2.943 ± 0.001	3.856 ± 0.761	1.31

Data are expressed as mean ± SD.

* Metabolite statistically significant (p<0.005). ULOQ: under limit of quantitation

### Mitophagy-deficient iPSC colonies exhibit a significantly reduced teratoma-initiating capacity

**Figure 6:**
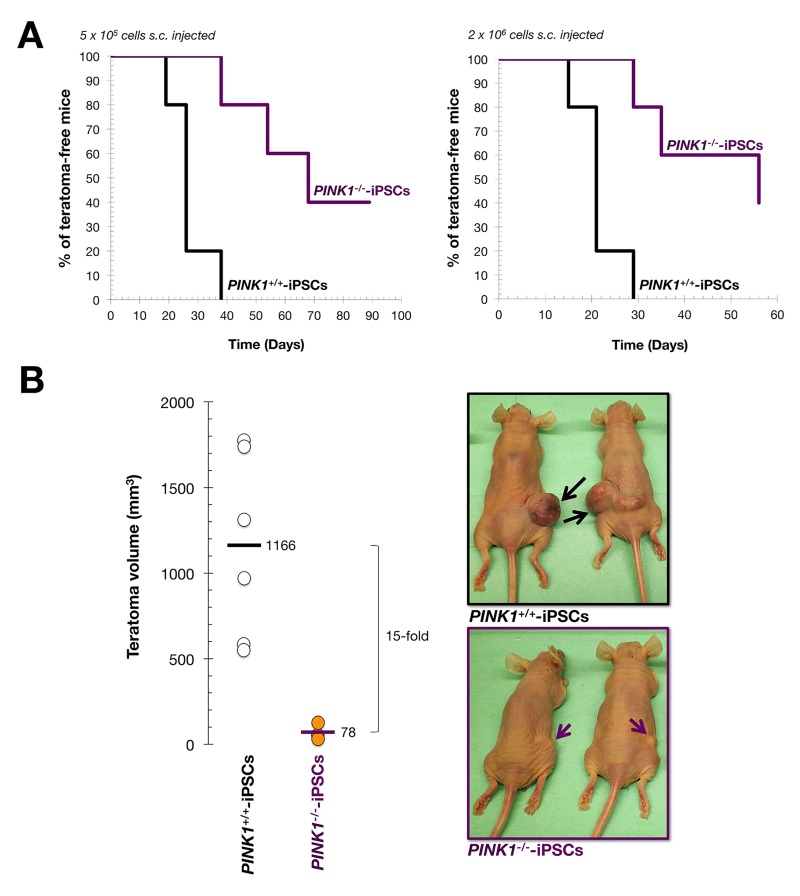
Mitophagy deficiency reduces tumorigenicity of iPSC colonies Athymic mice were injected with 5 × 10^5^ or 2 × 10^6^ cells obtained from *PINK1^+/+^*-iPSC or *PINK1^−/−^*-iPSC colonies. Teratoma growth rate was calculated by measuring teratoma volume. (**A**) Kaplan-Meier plots show the percentage of mice that remained teratoma-free after subcutaneous injection of iPSCs obtained as described in Fig. 2. (**B**) Mean teratoma volumes obtained at the end of the experiment (*left*) as well as representative images of animals bearing *PINK1^+/+^*-iPSC- and *PINK1^−/−^*-iPSC-derived teratomas after injection with 2 × 10^6^ cells (*right*). Note that teratomas formed from *PINK1^+/+^*-iPSCs present a dramatically higher growth rate that those derived from *PINK1^−/−^*-iPSCs.

The ability to derive the three germ layers generated during development, (ectoderm, mesoderm and endoderm), is the gold standard for determining whether potential iPSC candidates are fully pluripotent [78-80]. We thus examined the teratoma initiating and differentiation potential of *PINK1^+/+^*- and *PINK1^−/−^*-iPSCs *in vivo*. To exclude the possibility that any uncoupling of pluripotent capacity from tumorigenesis might be dose-dependent, i.e., with differentiation occurring at lower cell doses but tumors forming at higher cell doses, we injected 4- to 5-week-old athymic nude mice subcutaneously either with 5 × 10^5^ undifferentiated iPSCs or with 2 × 10^6^ cells, the latter being a saturating concentration to ensure the development of teratoma masses within a few weeks [64, 81]. We then analyzed efficiency, latency, and histology of teratoma composition. The rate of teratoma formation in *PINK1^+/+^*-iPSCs was 100% (6/6 mice in each group) regardless of the number of cells injected (Fig. 6A). In contrast, we observed a cell number-independent reduction in the rate of teratoma formation following the injection of *PINK1^−/−^*-iPSCs (60%; 4/6 mice in each group). Thus, the time required for 50% of animals to develop palpable teratomas was lengthened by 161% (from 26 to 68 days) upon injection of 5 × 10^5^ *PINK1^−/−^*-iPSCs, and by 166% (from 21 to 56 days) upon injection of 2 × 10^6^ *PINK1^−/−^*-iPSCs *in* (Fig. 6A). Indeed, injection of *PINK1^−/−^*-iPSCs resulted in drastically smaller teratomas than those observed upon injection of *PINK^+/+^*-iPSCs, i.e., the lesions in *PINK1^−/−^*-iPSC-injected mice were 15-fold smaller in size compared to the mean teratoma size observed in the *PINK^+/+^*-iPSC group (78 mm^3^ versus 1166 mm^3^, respectively) (Fig. 6B).

### Mitophagy-deficient iPSC colonies retain pluripotency and multi-germ layer differentiation potential

**Figure 7:**
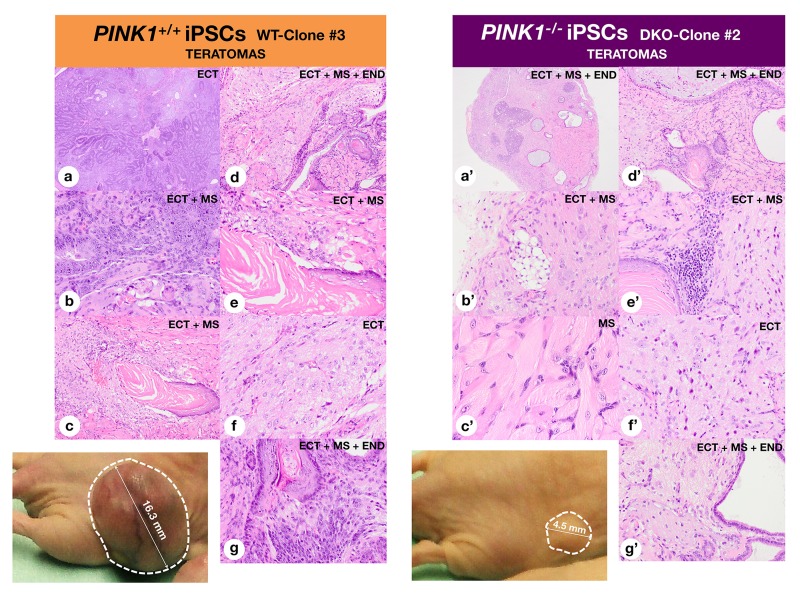
Mitophagy deficiency preserves the pluripotency of iPSCs Histological analyses of *PINK1^+/+^*- and *PINK1^−/−^*-iPSC-derived teratomas. Representative photographs of teratomas (circled with white dotted lines) are shown. *PINK1^+/+^*-iPSC-derived teratomas: (**a**) Primitive neural tissue, (**b**) Primitive neural tissue and skeletal muscle tissue, (**c**) Squamous epithelium, immature neural and glial tissue, (**d**) Mature nervous tissue, squamous keratinized epithelium, skeletal muscle tissue, mucinous glands, (**e**) Squamous keratinized epithelium, skeletal muscle tissue, (**f**) Mature nervous tissue, (**g**) Squamous keratinized epithelium, skeletal muscle tissue, respiratory epithelium. *PINK1^−/−^*-iPSC-derived teratomas; (**a'**) Whole-tumor section with dark areas of primitive neuroepithelium mixed with skeletal muscle tissue and seromucinous glands, (**b'**) Mature nervous tissue, (**c'**) Skeletal muscle tissue, (**d'**) Mature nervous tissue, squamous keratinized epithelium, skeletal muscle tissue, mucinous glands, (**e'**) Mature nervous tissue, squamous keratinized epithelium, skeletal muscle tissue, (**f'**) Mature nervous tissue, (**g'**) Mature nervous tissue, mucinous glands, osteoid substance. Note that teratomas from *PINK1^+/+^*-iPSCs and *PINK1^−/−^*-iPSCs similarly show mixed tissues apparently derived from the three germ layers, i.e., ECT: Ectoderm, MS: Mesoderm, and END: Endoderm.

Given the above results, it might be argued that blockade of teratoma formation upon loss of PINK1-dependent mitophagy was due to the failure of *PINK1^−/−^*-iPSCs to differentiate into primitive tissues representing all three germ layers. To question this, we carried out an ultrastructure analysis of teratomas from both groups of mice. Despite the lower efficiency in teratoma formation and the longer latency, tissue composition of *PINK1^−/−^*-teratomas was not noticeably different from equivalent *PINK^+/+^*-teratomas at the histological level. Hematoxylin-eosin staining showed various tissue derivatives of the three germ layers, including neural rosettes (ectoderm), gut-like epithelial tissues (endoderm), and smooth muscle, adipocytes, bone, and cartilage (mesoderm), in both teratoma groups (Fig. 7), confirming the full pluripotency and multi-germ layer differentiation potential of the iPSCs regardless of the PINK1-mediated mitophagy status. These findings, together with the fact that *PINK1^−/−^*-iPSCs normally expressed pluripotent markers confirmed that mitophagy-deficient iPSCs re-established pluripotency at the molecular and cellular level.

**Figure 8:**
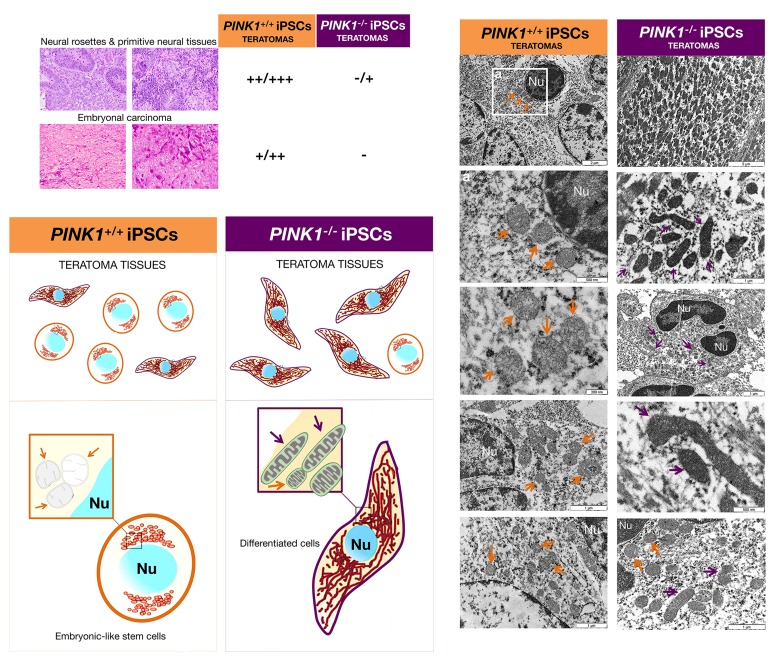
Mitophagy-deficient iPSC colonies are prone to direct differentiation *in vivo* *Left*. Analyses of histopathological features associated with the malignant behavior of iPSC-derived teratomas including the presence of neural rosettes/primitive neural tissues and embryonal carcinoma. Note that small *PINK1^−/−^*-iPSC-derived teratomas lack all the malignant features of iPSCs, which were highly abundant in *PINK1^+/+^*-iPSC-derived teratomas. “-“ means that no features are present, “+” a small number present, “++” a medium number, and “+++” a large number. *Right*. Representative TEM images of mitochondria in *PINK1^+/+^*- and *PINK1^−/−^*-iPSC-derived teratomas. While the mitochondria in many tissue sections from *PINK1^+/+^*-iPSCs-derived teratomas were characterized by a punctate, perinuclear arrangement, an electron-lucid matrix and poorly developed cristae, mitochondria in the majority of tissue sections from *PINK1^−/−^*-iPSC-derived teratomas formed more developed networks, had an electron-dense matrix and developed cristae. (Nu: Nucleus).

Although *PINK1^+/+^*- and *PINK1^−/−^*-iPSCs gave rise to teratomas composed of various recognizable tissue elements, we observed striking differences in the embryonal carcinoma (EC)-like component of poorly differentiated, primitive-appearing, blast-like teratocarcinoma stem cells. Accordingly, the large teratomas originating from *PINK1^+/+^*-iPSCs displayed extensive areas of undifferentiated tissue, e.g., abundant embryonic-appearing neuroepithelium with a high number of mitotic figures, which were considered as malignant based on the examination by a pathologist (Fig. 7). Conversely, *PINK1^−/−^*-iPSCs developed teratomas consisting almost exclusively of fully committed adult tissues, forming very small, morpho-logically benign, mature, and well-differentiated cystic lesions. Using TEM, we found that the cytoplasm of the extensive undifferentiated regions in *PINK1^+/+^*-iPSC-generated teratomas exhibited a simple architecture typical of embryonic-like cells in the early stages of development. Consequently, these regions were devoid of most organelles except for the presence of numerous ribosomes, a well-developed Golgi apparatus, and rough endoplasmic reticulum (Fig. 8). These regions contained few mitochondria and, when found, presented a globular shape with poorly developed cristae and electron-lucid matrix, and perinuclear localization, all indicative of functionally immature mitochondria. Conversely, tera-tomas from *PINK1^−/−^*-iPSCs generated tissues with conspicuous and numerous mitochondria, possessing a complex morphology with well-developed cristae, denser matrix, and elongated or branched appearance (Fig. 8).

Although it might be argued that, because mitophagy-deficient iPSC colonies tended to rapidly differentiate *in vitro* and exhibited a significantly reduced teratoma-initiating capacity, the percentage of fully reprogrammed cells might be significantly lower within *PINK1^−/−^*-iPSC colonies, these findings are also consistent with a more rapid differentiation of mitophagy-deficient iPSCs *in vivo*.

## DISCUSSION

Here we provide the first demonstration that mitophagy is a necessary mechanism for the conversion of somatic cells to a pluripotent cell fate with maximum efficiency. Our discovery that mitophagy-driven mitochondrial rejuvenation is required for induction and maintenance of stem cell pluripotency and that the mitophagy pathway plays a critical mitochondrial switch that determines the efficiency and quality of somatic reprogramming, illustrates how mitophagy can play a pivotal role in stem cell functions during aging and tissue regeneration.

**Figure 9:**
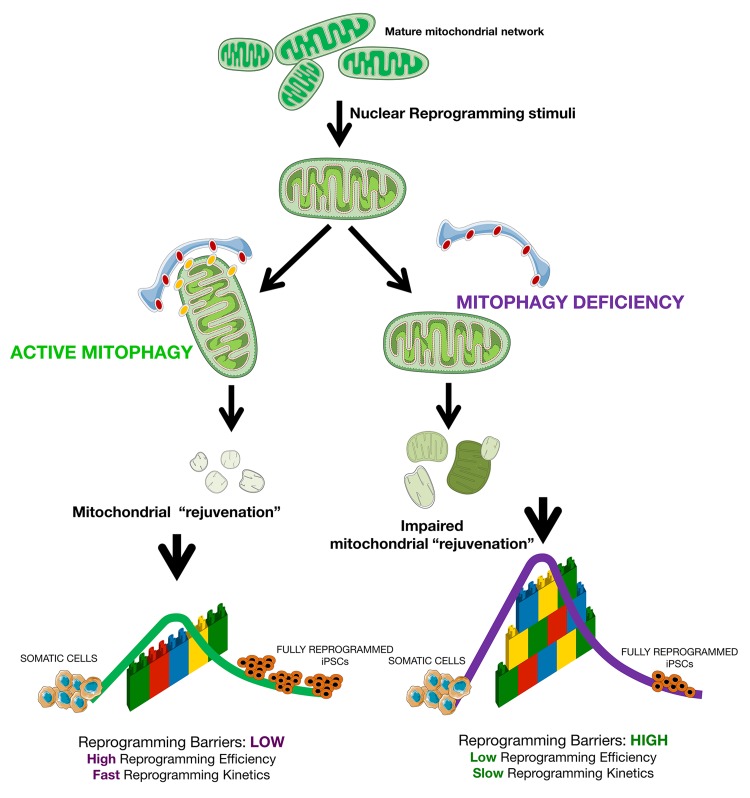
Mitophagy-regulated nuclear reprogramming of somatic cells into pluripotent stem cells Mitophagy is part of the roadmap during nuclear reprogramming of somatic cells to pluripotency and, as such, its blockade is sufficient to dramatically alter the speed and efficiency of iPSC reprogramming by “elevating” the “reprogramming barriers” of the epigenetic landscape and decreasing the size of the stem cell state basin of attraction, which results in the deceleration (i.e., lower efficiency and slower kinetics) of the nuclear reprogramming process. This conceptual figure represent cells stabilized in an initial non-pluripotent, somatic attractor and how nuclear reprogramming can make cells exceed the “reprogramming barriers”, represented as a wall of interlocking bricks, easier or harder in the presence or absence of PINK1-dependent mitophagy, respectively, and fall down in a final attractor of fully reprogrammed, induced pluripotent stem cell states. The cellular reprogramming process is presented as a colored line from the initial to the final cellular state.

We first addressed the question of whether mitophagy is a crucial process during nuclear reprogramming. Our findings reveal that the sole loss of PINK1-dependent mitophagy was sufficient to dramatically decrease the efficiency (~80% reduction) and speed of the nuclear reprogramming process (Fig. 9). Deficiency of PINK1-regulated mitochondrial quality control constitutes a previously unrecognized barrier to reprogramming. This fact, taken together with recent studies showing that whereas activation of DRP1-driven mitochondrial fragmentation contributes to the acquisition and maintenance of stem cell pluripotency [48, 50], deficiency of mitofusins Mfn1 and Mfn2 (which co-ordinately regulate mitochondrial fusion) instead elicits mitochondrial metabolic reprogramming to pluripotency [49], bolsters the notion that the ability of mitochondrial fission/fusion and mitophagy to restructure mito-chondrial dynamics is central for the control of cell-fate plasticity. Accordingly, it was reported that a restricted 2-day burst of autophagy (causing mitophagy) at the early stages of reprogramming was vital for iPSC generation [47].

We next focused on whether mitophagy is the driving mechanism for the conversion of functionally mature mitochondria to an immature state and vice versa, during reprogramming to stemness and commitment to terminal differentiation, respectively. The fact that loss of PINK1-dependent mitophagy impeded the full rejuvenation of the mitochondrial network during reprogramming indicates that mitophagy operates as a key mechanism in generating the immature mitochondrial structure commonly found in stem cells. Because a high level of mitophagy has recently been found to be a requisite for the high quality of mitochondria required for the stem cell state [82], and given that the number of AP^+^ colonies during early stages of reprogramming is used as an initial indicator of successful reprogramming of cells, the fact that cells within *PINK1^−/−^* iPSC colonies appeared to inherit a mixture of mature (“old”) and immature (“young”) mitochondria when compared with *PINK1^+/+^*-iPSCs colonies whose cells almost exclusively inherited immature mitochondria might suggest that the ratio of fully reprogrammed colonies among AP^+^ colonies is significantly lower in the absence of *PINK1*-dependent mitophagy. Furthermore, because AP expression levels is a less sensitive measure to differentiate between undifferentiated and early differentiating cells, the fact that mitophagy-deficient iPSC colonies normally expressed the pluripotent markers Oct4 and Sox2 together with their strong tendency to spontaneously differentiate and form heterogeneous populations of cells strongly suggest that PINK1-dependent mitophagy might be necessary for the iPSCs to remain in undifferentiated state. Indeed, with growing evidence for remodeling of energy metabolism in cell fate decisions, the fact that functional monitoring of cellular bioenergetics revealed an attenuated glycolytic capacity in mitophagy-deficient iPSC cells strongly suggest that the mitophagy pathway is an operating mechanism of mitochondrial switching that directs bioenergetic transition from somatic oxidative in somatic cells to glycolysis in iPSCs. While mitophagy might ultimately determine the efficiency and quality of nuclear reprogramming and stemness transition in somatic cells by participating in the bioenergetic conversion for establishing functional pluripotency, it remains to be unambiguously defined whether the ultimate role of mitophagy in stem cells is to regulate the preferential, asymmetric apportion of younger mitochondria during self-renewal [82].

The main characteristics of iPSC mitochondria are their rounded morphology with condensed cristae and their poor oxidative activity due to the low membrane potential [36-45, 65]. Given that mitophagy is triggered by mild oxidative stress in a mitochondrial fission-dependent manner [83], we hypothesized that abnormal mitophagy might lead to the accumulation of “old”, ROS-generating mitochondria, and this, in turn, might impair the efficiency of reprogramming. Our results showed that the mitophagy deficiency-imposed roadblock for reprogramming is bypassed, in part, by the ROS scavenger vitamin C upon the inclusion of oncogenic c-Myc, which is a key inducer of glycolytic reconfiguration [31, 84]. Interestingly, c-Myc also functions as the major contributor of reprogramming-mediated oxidative stress [38, 85]. Therefore, while it seems likely that ROS partially contribute to the lower reprogramming efficiency of *PINK1^−/−^* MEFs, other ROS-independent mitochondrial changes imposed by the loss of PINK1-driven mitophagy seem to operate as a dominant roadblock during reprogramming. Because histone demethylases have been shown to be the direct downstream effectors of vitamin C-dependent enhancement of cell reprogramming, in addition to its antioxidant activity [72-75], it is possible that mitophagy deficiency might impede reprogramming by inhibiting histone demethylation. In this regard, it was noteworthy that the iPSC metabolite majorly impacted by loss of *PINK1*-dependent mitophagy was α-ketoglutarate, a key mitochondrial metabolite that is siphoned from the TCA cycle to support rapid cell proliferation via lipid and amino acid biosynthesis that can exit also the mitochondria to function as a cofactor for dioxygenase enzymes including Jumonji-family histone demethylases, TET-family DNA hydroxylases, and prolyl hydroxylases [86-88]. Indeed, the intracellular α-ketoglutarate levels have been shown to contribute to the maintenance of cellular identity and have a mechanistic role in the transcriptional and epigenetic state of stem cells [89]. Further research, however, is needed to evaluate the precise epigenetic modifications associated with mitophagy-related changes in mitochondrial biogenesis, structure, and function, i.e., how mitophagy might drive the acquisition and maintenance of stemness by meta-bolically regulating the epigenetic landscape of the nuclear genome [90-92].

The fact that *PINK1^−/−^*-iPSCs possessed a normal capacity to differentiate into mature tissue confirmed that deficiency of PINK1-driven mitophagy does not interfere with the pluripotent quality of iPSCs. Because the kinetics of teratoma formation is dependent on the number of remaining pluripotent stem cells during the differentiation procedure [93-98], it might be tempting to suggest that loss of PINK1-driven mitophagy facilitates the depletion of residual undifferentiated pluripotent cells (teratocarcinoma-initiating cells) during *in vivo* teratoma formation. Moreover, because the retention of the embryonal character is considered the basis for continuous and progressive growth of malignant teratomas, loss of biological aggressiveness in *PINK1^−/−^*-iPSC-derived teratomas apparently suggest that mitophagy deficiency impedes the retention of undifferentiated pluripotent stem cells by promoting their differentiation and inhibiting potential dedifferentiation of committed cells. Accordingly, the highly significant more active mitochondrial state of tissues from *PINK1^−/−^* teratomas might be indicative of rapid, most committed differentiation of otherwise pluripotent *PINK1^−/−^*-iPSCs. Conversely, it might be argued that these findings reflect that the percentage of fully reprogrammed cells might be significantly lower within mitophagy-deficient *PINK1^−/−^*-iPSC colonies. Forthcoming studies should evaluate whether rapidly differentiating, mitophagy-deficient heterogeneous colonies of *PINK1* KO-iPSCs might illuminate new mitochondria-centered mechanisms aimed to restore or stimulate a differentiation checkpoint capable of limiting the aberrant self-renewal of life-threatening cancer stem cells in tumor tissues.

We are beginning to dissect the roles of mitochondria in the establishment and homeostasis of stemness, which may help to uncover novel insights into our understanding of a wide variety of degenerative diseases, aging, and aging-related diseases including cancer. Although we are still far from a comprehensive understanding of the physiological functions of mitochondria in stem cells, our findings extend previous studies into the causal mechanism behind the well-recognized metabolic switch during the establishment of pluripotency, which is accompanied by significant changes in mitochondrial function, composition, structure, maturation, and signaling. Mitophagy appears to be a crucial cellular process for the conversion of functionally mature mitochondria to an immature state and vice versa during reprogramming and differentiation, respectively. In this regard, mitophagy may ensure a metabolic transition to meet the specific energetic and anabolic demands of the stemness state, e.g., mitophagy-induced repression of mitochondrial functions including mitochondrial clearance might accelerate the onset of the glycolytic metabolism [99]. Furthermore, mitophagy-driven mitochondrial rejuvena-tion might contribute to the ability of stem cells to suppress differentiation by orchestrating the mitochondria function as signaling organelles of diverse biological functions [100, 101], including not only bioenergetic transitions but, perhaps more importantly, metabolome remodeling traits connecting mitochondrial metabolites with epigenetics [100-102]. Further studies are warranted to determine the causal role of mitophagy-driven mitochondrial rejuvenation as part of the mechanism involved in the maintenance and asymmetric transmission of the pluripotency and differentiation fate of stem cells. Our discovery that mitophagy-controlled mitochondrial quality is a critical director of cell-fate plasticity and stem-cell fate should provide new insights into how mitophagy might influence the stem cell decisions to retain pluripotency or differentiate in tissue regeneration and aging, tumor growth, and regenerative medicine.

## MATERIALS AND METHODS

### *PINK1*-knockout mouse embryonic fibroblasts

*PINK1*-knockout mice were generated by targeted deletion of exon 1 as described [52]. Loss of Pink1 mRNA expression in primary embryonic fibroblasts (MEFs) was confirmed by quantitative RT-PCR [52].

### Generation of iPSCs

Mouse primary iPSCs were created by transducing MEFs deficient for *PINK1* (*PINK1^−/−^)* and wild-type (*PINK1^+/+^)* counterparts with the pMXs-based retroviruses that individually encode the mouse transcription factors Oct3/4, Sox2, and Klf4 following a previously-described protocol [64, 65]. Characterization of iPSC-like colonies was carried out by analyzing pluripotent marker expression by alkaline phosphatase (AP) staining using the StemTAG™ Alkaline Phosphatase Staining and Activity Assay Kit (Cell Biolabs, Inc. Cat. No. CBA-302) and the expression of Oct3/4, Nanog, Sox2, and Ssea-1 by immunofluorescence (see below).

To generate feeder-free iPSC cultures for teratoma assays, culture plates were coated with 0.3 mg/mL Matrigel (growth factor-reduced, BD Biosciences, San Jose, CA) at 4°C overnight. Unbound Matrigel was aspirated, and the cells were washed with DMEM/F12 medium. iPSCs were seeded on Matrigel-coated plates in MEF-conditioned ES cell medium supplemented with leukemia inhibitory factor (LIF) and bFGF (4 ng/mL). The medium was changed every day.

### Immunofluorescence staining

High-content confocal imaging was performed in 96-well clear bottom imaging tissue culture plates (BD Biosciences) optimized for automated imaging applications. Triton^®^ X-100 permeabilization and blocking, primary antibody staining, secondary antibody staining using Alexa Fluor^®^ 488 goat anti-rabbit/mouse IgG (Invitrogen, Molecular Probes, Eugene, OR) and counterstaining (Hoechst 33258; Invitrogen) were performed following BD Biosciences protocols. Images were captured in different channels for Alexa Fluor® 488 (pseudocolored green) and Hoechst 33258 (pseudocolored blue) on a BD PathwayTM 855 Bioimager System (BD Biosciences) with 20× or 40× objectives (NA 075, Olympus). Merged images were obtained according to the Recommended Assay Procedure using BD Attovision^TM^ software.

### Reactive Oxygen Species (ROS) detection

Cells were incubated for 60 min with 10 μmol/L 2′,7′-dihydro-dichlorofluorescein-diacetate (H2DCF-DA) (Invitrogen, Molecular Probes) at 37˚C. Cellular green fluorescence was then measured by flow cytometry. Cell-permeant non-fluorescent H_2_DCF-DA, upon cleavage of the acetate moiety by intercellular esterases and oxidation by ROS, is converted to strongly fluorescent DCF and thus reports the ROS abundance.

### Immunoblotting

Equal concentration of proteins (50 μg) was loaded into a 10% SDS-polyacrylamide gel and then electrotransferred. After blocking (5% nonfat powder milk in TBS plus 0.1% TritonX100 for 1 h at room temperature), the nitrocellulose membranes were incubated for 16-20 h at 4°C with the primary antibody (Oct3/4, Abcam ab-1985, 1:600; Sox2, Abcam ab-97959, 1:1000; β-actin, Santa Cruz sc-47778; 1:500). The detection of the immune complexes after incubation with the appropriate peroxidase-conjugated secondary antibody (Cell Signaling #7074, 1:1000; Calbiochem #401215, 1:5000) was performed with the Clarity™ Western ECL Substrate (Bio-Rad).

### Extracellular flux bioenergetic assays

Extracellular acidification rates were measured using an XF Extracellular Flux Seahorse Analyzer (Seahorse Bioscience). XFp Glycolysis Stress tests were performed in accordance with manufacturer's instructions. Each plotted value is the mean of at least 6 replicates and was normalized to Hoechst signal in each well.

### Targeted metabolomics and data analysis

Measurements of bioenergetics metabolites obtained from *PINK1^+/+^*-iPSC and *PINK1^−/−^*-iPSC clonal cell lines were performed by employing a previously described simple and quantitative method based on gas chromatography coupled to quadrupole-time of flight mass spectrometry and an electron ionization interface (GC-EI-QTOF-MS) [76, 77].

Raw data were processed and compounds were detected and quantified using the Qualitative and Quantitative Analysis B.06.00 software (Agilent Technologies), respectively. MetaboAnalyst 3.0 ( http://www.metaboanalyst.ca) was used to generate scores/loading plots, Heatmaps, and correlation maps [103].

### Teratoma assays

To form teratomas, iPSCs were harvested from Matrigel-coated culture dishes and injected subcutaneously (s.c.) into the dorsal flank of female athymic nude mice (four- to five-weeks-old, 23-25g; Harlan Laboratories, France). Mice were weighed once per week. Teratomas were measured daily with electronic calipers and tumour volumes were calculated using the formula: volume (mm3) = length × width^2^ × 0.5. General health of the mice in response to teratoma development (e.g., subcutaneous teratomas cause ulceration on the skin) was monitored daily by a specialized veterinarian. Teratomas were carefully dissected and removed in entirety, fixed in 10% phosphate buffered formalin (3.6% formaldehyde) for 24 hours, and paraffin-embedded. For histopathological analysis, consecutive sections (4 μm) were cut and stained with haematoxylin and eosin according to standard procedures.

The Institutional Animal Care and Use Committee (IACUC) of the Institut d'Investigació Biomèdica de Bellvitge (IDIBELL; Animal Use Protocol #6302 authorized by the Animal Experimental Commission from the Catalan Government, Barcelona, Spain) approved the experiments.

### Transmission electron microscopy

Small pieces of teratomas were fixed in a 2% glutaraldehyde solution in 0.1 M cacodylate buffer, pH 7.4. Samples were then post-fixed in 1% osmium tetroxide (OsO_4_) for 2 h and dehydrated through a graded series of acetone prior to impregnation in increasing concentrations of resin in acetone over a 24 h period. Semi-thin sections (500 nm) were stained with 1% toluidine blue. Ultrathin sections (70 nm) were subsequently cut using a diamond knife, double-stained with uranyl acetate and lead citrate, and examined with a transmission electron microscope (Hitachi, Tokyo, Japan).

### Statistical analysis

The results are presented as the mean ± SD of at least three repeated individual experiments for each group. The analyses were performed using XLSTAT 2010 (Addinsoft^TM^). A P-value ≤ 0.05 was considered statistically significant.

## SUPPLEMENTAL DATA FIGURES


