# Supplementary material for: Mitophagy-driven mitochondrial rejuvenation regulates stem cell fate
Source: Aging (Albany NY). 2016 Jun 13;8(7):1330–49. doi: 10.18632/aging.100976 (PMC4993334; doi:10.18632/aging.100976)
Supplement: Supplementary file 1 [file aging-08-1330-s001.pdf]

SUPPLEMENTAL DATA

Loss of PINK1 prevents mitophagic elimination of damaged mitochondria

To test whether *PINK1*-KO mouse embryonic fibroblasts (MEFs) constitute a useful model to dissect the role of mitophagy in the establishment of induced pluripotency, we first mimicked mitochondrial damage by experimentally depolarizing mitochondria [62]. To probe PINK1-dependent autophagic digestion of mitochondria (Supplemental Fig. 1A), we tracked the intracellular distribution of MitoTracker in $PINK^{+/+}$ and $PINK^{-/-}$ MEFs upon treatment with the uncoupler carbonyl cyanide *m*-chlorophenylhydrazone (CCCP), a lipid-soluble weak acid that drastically increases proton permeability across the mitochondrial inner membranes, thus dissipating the transmembrane potential and depolarizing the mitochondria. Because MitoTracker becomes covalently bound to mitochondrial proteins after uptake and remains in mitochondria even if a mitochondrion subsequently depolarizes, loss of MitoTracker after mitophagy stimulation largely represents lysosomal digestion of mitochondrial peptide fragments or breakage of covalent bonds between MitoTracker and mitochondrial proteins [67].

When we monitored mitochondrial clearance following treatment with 20 μmol/L CCCP for 18 h (Supplemental Fig. 1B, *top panels*), no MitoTracker-labeled mitochondria were detected in 86±6% of $PINK1^{+/+}$ MEFs (Supplemental Fig. 1C). In clear contrast, mitochondria were retained in *PINK1*-deficient cells (Supplemental Fig. 1B, *bottom panels*) and mitochondrial clearance occurred solely in a very small percentage (14±7%) of $PINK1^{-/-}$ MEFs following depolarization with CCCP (Supplemental Fig. 1C).

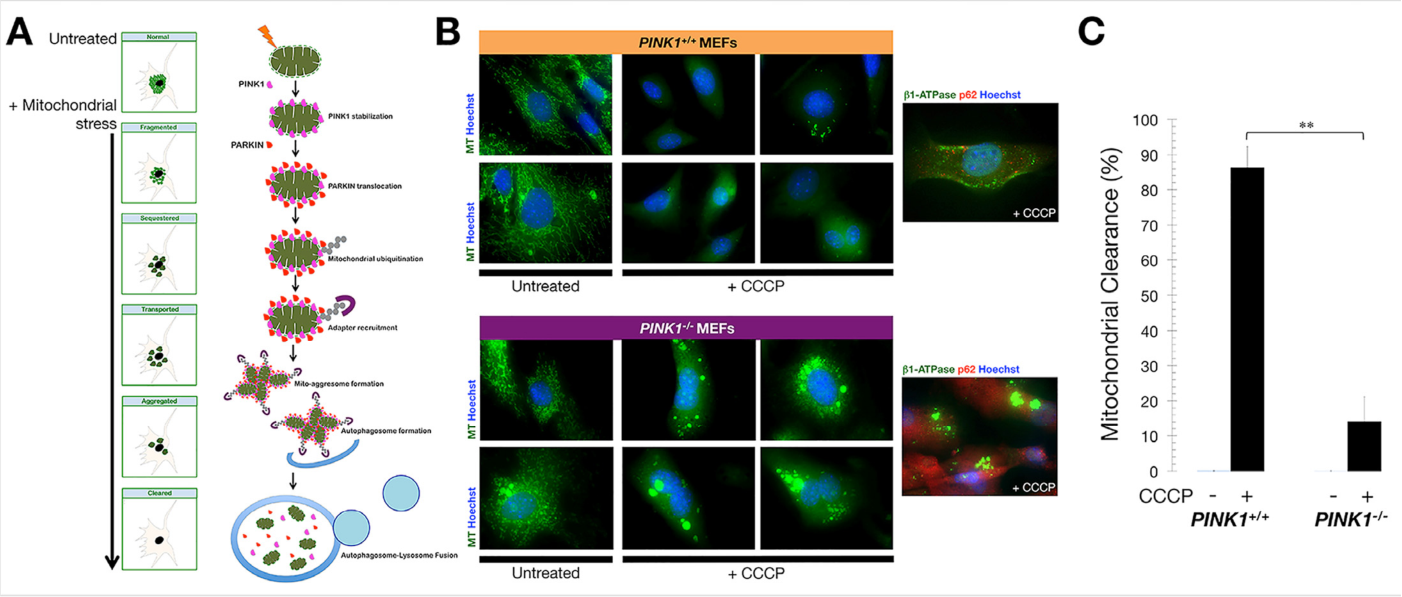

Supplemental Figure 1. PINK1 is necessary to drive mitophagy. (**A**) The schematic illustrates individual steps of the apparently sequential process of PINK1-dependent mitophagy. In response to several mitochondrial stresses, including chemical uncoupling (by CCCP treatment), mtDNA mutations, increasing ROS levels, and other yet-to-be discovered stimuli (e.g., nuclear reprogramming), PINK1 becomes stabilized on the mitochondrial outer membrane of unwanted or damaged mitochondria. Association of PINK1 with mitochondria results in the subcellular distribution and translocation of the ubiquitin ligase Parkin, which ubiquitinates specific mitochondrial surface proteins, such as mitofusins (Mfn1/2). Mitochondrial ubiquitination, in turn, results in the recruitment of adapter proteins including p62/SQSTM1 and HDAC6 that ultimately links ubiquitinated substrates to cellular transport and autophagic machineries. This transport results in the peri-nuclear clustering and formation of mito-aggresomes, which may thereby serve to restrict cellular distribution of unwanted or damaged mitochondria as well as to prepare targeted mitochondria for autophagic clearance. LC3 and autophagosomal membranes are co-recruited during transport and aggregation of targeted mitochondria and, consequently, mito-aggresomes become engulfed by the forming autophagosomes that finally fuse with lysosomes for degradation and mitochondria clearance. (**B**) $PINK1^{+/+}$ MEFs (*top*) and $PINK1^{-/-}$ MEFs (*bottom*) were preloaded with MitoTracker followed by an 18 h treatment with 20 μmol/L (μM) CCCP (MT: MitoTracker). Cells were then fixed (MitoTracker Red is retained after permeabilization) and, where shown, immunostained with an antibody against the β1-subunit of the mitochondrial F1-ATPase to visualize mitochondria or an antibody against p62. **C.** Average percentages of mitochondria-free cells from **A** (n=3). **, P<0.01.

Indeed, CCCP treatment resulted in the formation of large MitoTracker-positive aggregates in *PINK1*$^{-/-}$ MEFs, mainly in the perinuclear region, with relatively sparse cytosolic staining (Supplemental Fig. 1B, *bottom panels*). We further confirmed this mitophagy defect by immunostaining with an antibody against the β1-subunit of the mitochondrial F1-ATPase complex, which strongly labeled mitochondrial aggregates in *PINK1*$^{-/-}$ MEFs (Supplemental Fig. 1B). Similarly, staining of p62/SQSTM1, an adapter protein that binds ubiquitinated mitochondria, was readily detectable in *PINK1*$^{-/-}$ MEFs, consistent with the accumulation of mitochondria.

Mitophagy deficiency-imposed roadblock for reprogramming is partially circumvented by vitamin C.

Using 2'-7'-dichlorodihydrofluorescein diacetate (DCFH-DA) to measure ROS generation in the cytoplasm and cellular organelles such as the mitochondria, we noted a marked increase in oxidative stress in mitophagy-deficient *PINK1*$^{-/-}$ MEFs relative to mitophagy-competent *PINK1*$^{+/+}$ MEFs following treatment with CCCP (Supplemental Fig. 2A). Indeed, although an inspection of the temporal production of

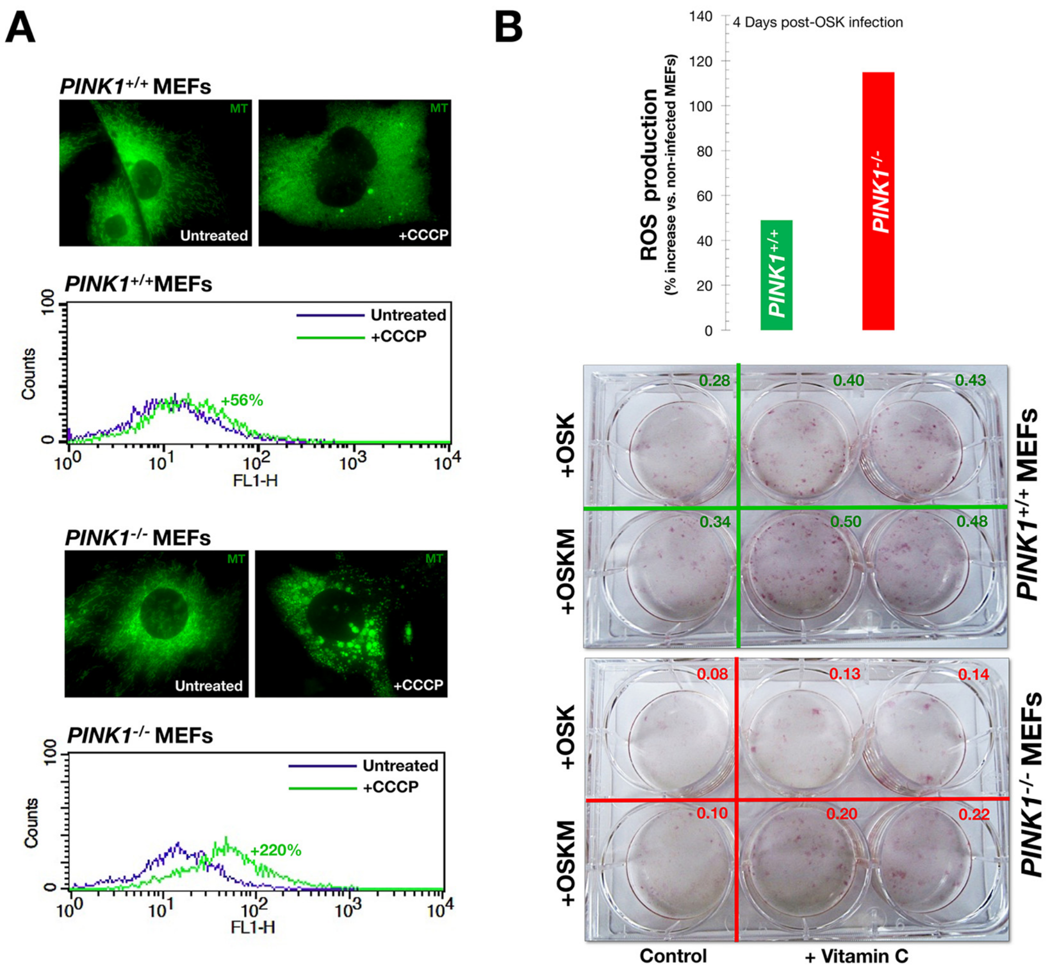

Supplemental Figure 2. Mitophagy deficiency enhances oxidative stress in response to reprogramming. (**A**) MitoTracker was used to stain mitochondria in live *PINK1*$^{+/+}$ and *PINK1*$^{-/-}$ cells after short-term (1 h) treatment with CCCP (*top*). Measurement of ROS in CCCP-treated cells (*bottom*). Untreated or CCCP-treated *PINK1*$^{+/+}$ and *PINK1*$^{-/-}$ MEFs were exposed for 60 min to H_2DCF-DA and fluorescence intensity was measured by flow cytometry. Shown are representative scatter plot histograms of DCF fluorescence. (**B**) Measurement of ROS in OSK-transduced cells (*left*). Bar charts of representative measurements of ROS levels (% increase versus baseline ROS levels in untransduced MEF populations cultured in parallel) in *PINK1*$^{+/+}$ MEFs (*top*) and *PINK1*$^{-/-}$ MEFs (*bottom*) at days indicated post-infection with OSK. **C.** Representative AP staining of OSK- and OSKM-infected untreated or treated with vitamin C (*right*). For each experimental condition, reprogramming efficiency was calculated by dividing the average count of AP-positive colonies per well by the initial number of cells plated (n=6 for each condition).

ROS during reprogramming revealed that both cell types displayed a marked burst of ROS at day 4 after OSK transduction, ROS production at this time was significantly greater in *PINK1*$^{-/-}$ MEFs compared with equivalent wild-type cells (Supplemental Fig. 2B, *top*).

A testable prediction from this observation is that inhibition or alleviation of excessive ROS production should increase the number of mitophagy-deficient cells that surpass the early reprogramming barrier imposed by abnormal mitophagy. We therefore evaluated whether the ability of vitamin C to block steady-state ROS levels in OSK-transduced *PINK1*$^{-/-}$ MEFs might lead to an increase in reprogramming efficiency. Addition of vitamin C (50 μg/mL) notably increased the reprogramming efficiency of OSK-transduced *PINK1*$^{-/-}$ MEFs (Supplemental Fig. 2B, *bottom*); however, when both cell types were supplemented with vitamin C during reprogramming, OSK-transduced *PINK1*$^{+/+}$ MEFs still generated significantly more colonies than *PINK1*$^{-/-}$ MEFs (Supplemental Fig. 2B, *bottom*).
